# Digital analysis of the human maxilla to enable semistandardized template tool reconstructions with free fibula transplants

**DOI:** 10.1007/s00784-024-05908-8

**Published:** 2024-09-07

**Authors:** Christopher-Philipp Nobis, Clara Kübler, Manuel Olmos, Katja Schulz, Jacek Glajzer, Joy Backhaus, Ragai Matta, Marco R. Kesting, Rainer Lutz

**Affiliations:** 1 Department of Oral and Cranio-Maxillofacial Surgery, University Hospital Erlangen, Friedrich-Alexander-Universität Erlangen-Nürnberg, Glueckstrasse 11, 91054 Erlangen, Germany; 2 Institute of Medical Teaching and Medical Education Research, University Hospital of Würzburg, Würzburg, Germany; 3 Department of Prosthodontics, University Hospital Erlangen, Friedrich-Alexander-Universität Erlangen-Nürnberg, Erlangen, Germany

**Keywords:** Maxillary reconstruction, Head and neck anatomy, Reconstructive surgery, Digital morphology analysis, Fibula osteotomy, Microvascular fibula transplant

## Abstract

**Objectives:**

This study analyzed the human maxilla to support the development of mean-value-based cutting guide systems for maxillary reconstruction, bridging the gap between freehand techniques and virtual surgical planning (VSP).

**Materials and methods:**

This retrospective cohort study used routine CT scans. DICOM data enabled 3D modelling and the maxilla was divided into four regions: paranasal (R1), facial maxillary sinus wall (R2), zygomatic bone (R3) and alveolar process (R4). Surface comparisons were made with a reference skull. Statistical analyses assessed anatomical variations, focusing on mean distance (Dmean), area of valid distance (AVD), integrated distance (ID) and integrated absolute distance (IAD). The study addressed hemimaxillectomy defects for two-segmental reconstructions using seven defined bilateral points to determine segmental distances and angles.

**Results:**

Data from 50 patients showed R2 as the most homogeneous and R4 as the most heterogeneous region. Significant age and gender differences were found in R3 and R4, with younger patients and females having more outliers. Cluster analysis indicated that males had R1 and R3 positioned anterior to the reference skull. The mean angle for segmental reconstruction was 131.24° ± 1.29°, with anterior segment length of 30.71 ± 0.57 mm and posterior length of 28.15 ± 0.86 mm.

**Conclusions:**

Anatomical analysis supported the development of semistandardized segmental resection approaches. Although gender and anatomical differences were noted, they did not significantly impact the feasibility of mean-value-based cutting-guide systems.

**Clinical relevance:**

This study provides essential anatomical data for creating cost-effective and efficient reconstruction options for maxillary defects, potentially improving surgical outcomes and expanding reconstructive possibilities beyond current techniques.

## Introduction

Although now common practice in head and neck surgery, bony reconstruction of the maxilla using microvascular grafts remains a challenge for reconstructive surgeons.

The free fibula transplant (FFF) has proven to be a particularly suitable workhorse for jaw reconstruction in oral and maxillofacial surgery due to its anatomical characteristics, reported low rates of flap failure, and relatively low donor site morbidity [1]. The osteomyocutaneous flap provides three tissue qualities, bone, muscle and skin, which can be used specifically according to the reconstructive requirements. However, challenging anatomical anomalies of the flap skin vessels or the leg arteries can also be present [2]. A key advantage is the flap’s reconstructive flexibility, allowing free placement of different segmental osteotomies and the harvesting of two separate skin islands for combined intra- and extraoral defect closure [3]. This facilitates large and complex reconstructions with multiple segments [4]. Additionally, FFF reconstructions support dental rehabilitation through the placement of dental implants [5, 6], with established mono-barrel graft concepts for both lower and upper jaw rehabilitation [7]. Dental implants are often the only option for adequate dental rehabilitation after the loss of large bony sections of the jaw, as removable, mucosa-supported restorations often cannot be adequately fixed to the newly reconstructed soft tissue [8]. The jaw is also highly sensitive to factors such as facial harmony, chewing, airway patency and speech [9]. Therefore, accurate preoperative planning is necessary to achieve predictable reconstructive results.

For complex cases of maxillary defects, the use of virtual surgical planning (VSP) has become a common solution to this task, especially for segmental maxillary reconstructions. VSP can facilitate segmental osteotomies, thereby increasing surgical accuracy and providing optimal reconstruction for subsequent dental rehabilitation [10]. The main disadvantages of VSP are increased financial costs, a lack of intraoperative flexibility and the need for more extensive preoperative preparation. In less complex cases, the conservative procedure of reconstruction is usually performed with intraoperative osteotomies and segmental adjustments according to the size of the defect. This requires a high level of surgical expertise and experience. There is currently no intermediate solution for maxillary bone reconstruction between VSP and the conservative procedure. In the mandible, an alternative surgical technique has been successfully developed with the introduction of partially adjustable cutting guide systems [11]. These systems are suitable for the reconstructive problems of medium difficulty and, with the right indication, can achieve good results compared to VSP [12]. In the maxillary region, devices of similar quality are not yet available. The development of a partially adjustable cutting guide system for the mandible requires a prior analysis of the anatomy in terms of relevant reconstructive parameters. In the mandible, the possibility of developing such a device could be demonstrated by such an analysis [13]. No relevant data or prior analysis could be found in the available literature for the maxilla. This may be due to the fact that the anatomy of the maxilla is spatially more complex and therefore the surface analysis is much more extensive. Thus, the aim of this study was to provide missing data and to analyze the anatomy of the human maxilla with a view to the possible development of a partially adjustable cutting guide system analogous to previous work in the mandible.

## Materials and methods

### Study design and data acquisition

A retrospective cohort study was conducted to evaluate the anatomy of the maxillary region in relation to the development of partially adjustable cutting guide systems. Data collection and further analysis were approved by the local ethics committee of the Friedrich-Alexander-Universität Erlangen-Nürnberg (registration number 18_21 B). The study was conducted in accordance with the tenets of the Declaration of Helsinki.

### Study sample

This study included high-resolution computed tomography (CT) scans of 50 patients, all treated at the Department of Oral and Cranio-Maxillofacial Surgery at the University Hospital Erlangen. CT scans from routine diagnostics were screened until 50 scans meeting the study’s inclusion criteria were obtained. These scans were retrospectively retrieved from routine diagnostic procedures, with no additional radiological imaging required specifically for the study. All patients provided written consent for their CT images to be used for scientific purposes. To be included in the study, patients had to be at least 18 years old. Another inclusion criterion was the availability of a high-resolution, thin-slice CT scan of the skull. Exclusion criteria for the study were an age under 18 years, lack of capacity to consent, lack of care and bony defects in the region of interest.

### 3D-Model development

The radiological data of the enrolled patients were acquired as DICOM files, and relevant clinical data were also obtained. All the data were then anonymized and imported into the software *Mimics Innovation Suite* software (MIS 20.0, Materialise, Leuven, Belgium) for 3D modelling. The soft tissue could be excluded in a virtual dissection by setting a threshold for the Houndsfield units. The setting was adjusted from 226 HU to a maximum of 48,060 HU, and then the selection was adjusted individually for each patient's skull [14]. The models were finalized and then exported as STL files [15]. Further model optimization with artefact removal and anatomical reconstruction of minor errors was performed using *Meshmixer* (software Autodesk Inc., Version 3.5.474, 2017).

### Reconstructive regions of interest

**Fig. 1:**
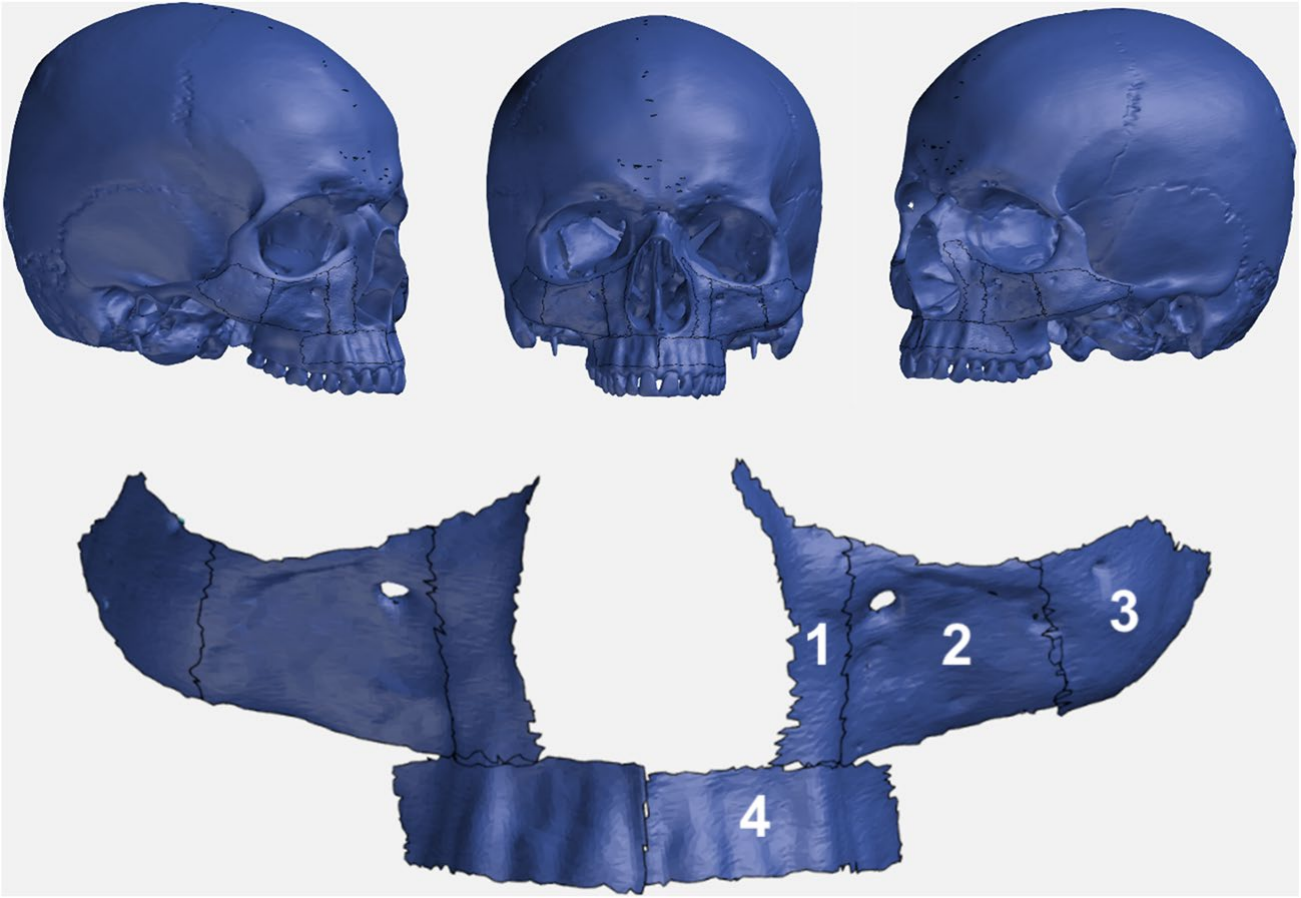
The four identified reconstructive regions of interest were: paranasal region (1), facial maxillary sinus wall (2), zygomatic bone (3) and alveolar process (4)

The maxilla has been divided into 4 regions of reconstructive interest for the development of partially adjustable cutting guide systems. Such a standardized system would not be suitable for the most complex reconstructive cases, but rather for more standardized defect situations, such as hemimaxillary defects with sufficient bony contact area for the placement of adjustable cutting guides. The focus was therefore focused on class I and II defects according to the Brown and Shaw classification [16]. The regions of interest were named according to their location as follows: region 1, paranasal; region 2, facial maxillary sinus wall; region 3, zygomatic bone; and region 4, alveolar process (Fig. 1).

### Anatomical analysis

**Fig. 2:**
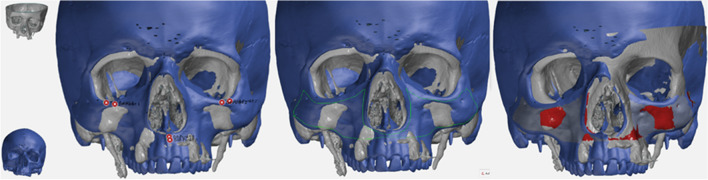
Superimposition of reference and patient skull via the 3-point alignment and the local best fit

**Fig. 3:**
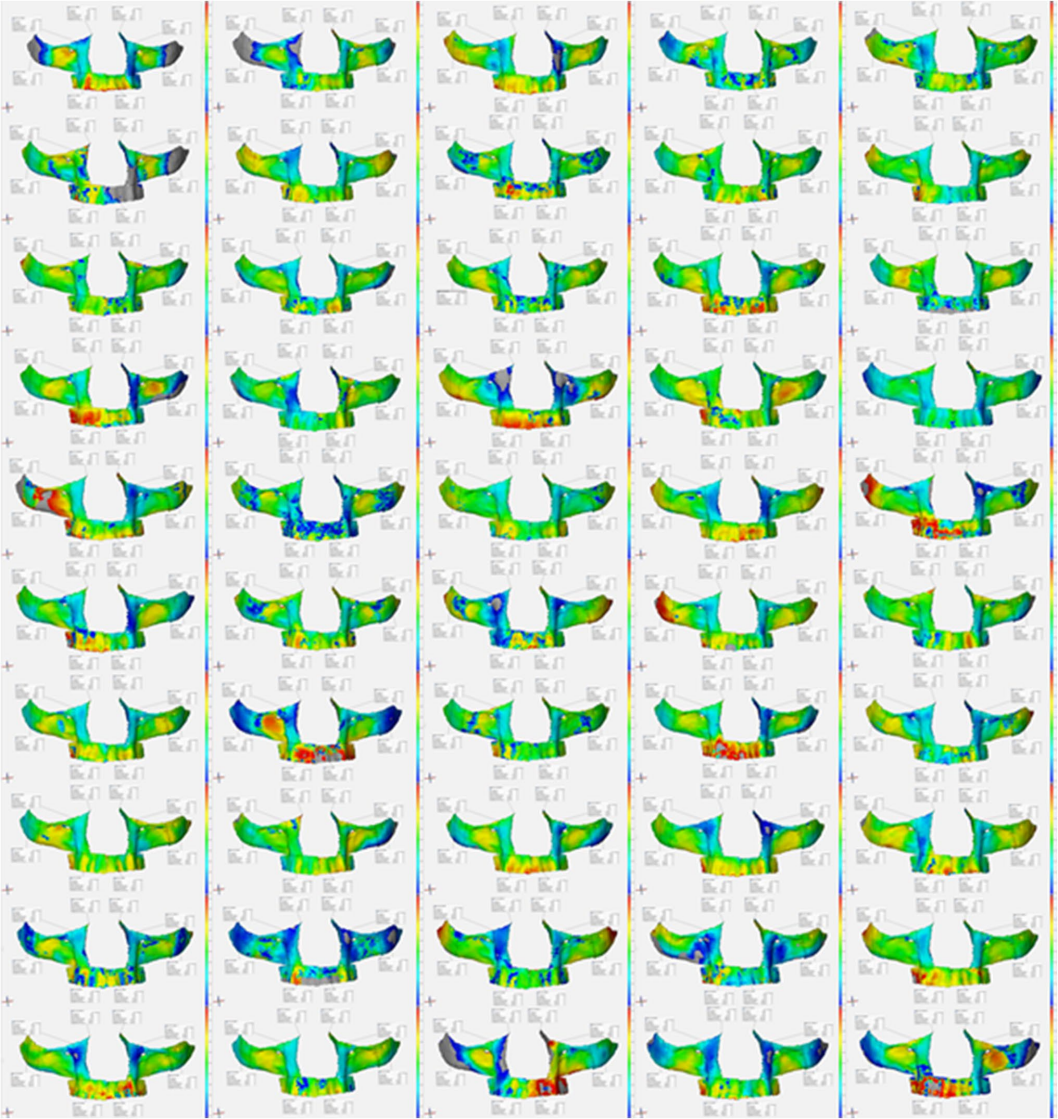
Overview of all 50 surface comparisons

A constant reference was needed to allow comparison between the patients’ 3D models. For this purpose, an artificial skull model (QS 1, SOMSO Modelle GmbH, Coburg, Germany) was used, on which a high-resolution CT scan was performed and a 3D model was created. The 3D model was imported into the *GOM Inspect* software (Build 2021–06-18, GOM GmbH, Braunschweig, Germany), and the reconstructed regions of interest were then manually marked. The defined surfaces were then converted to CAD format before surface comparisons were performed individually for all regions. All patient skulls were compared to the reference skull for surface and volume comparison. In order to best align the skulls, an initial 3-point alignment was performed using anatomically characteristic and constant points. These included the A-point and the deepest point of the inferior orbital rim (IOr/IOl point) on each side [17]. The software then superimposed the two skulls, and the primary alignment was selected using local best fit. This method optimizes the primary alignment by matching points within a radius of 4.0 mm [18]. For surface comparisons, a measurement distance of -5 mm anteriorly and + 5 mm posteriorly was used to record a total deviation of 1 cm. The distance between the skulls was visualized using a false color image (Fig. 2). Surface deviations are shown in colors ranging from blue to green to red relative to the position of the reference skull. Blue indicates areas in front, red indicates areas behind the reference skull, and deviations of less than 1 mm are shown in green (Fig. 3). In addition to the visual representation of the area comparisons, output values were generated for the following seven parameters for each region: maximum distance (Dmax), minimum distance (Dmin), mean distance (Dmean), distance standard deviation (DSD), area of valid distance (AVD), integrated distance (ID) and integrated absolute distance (IAD).

**Fig. 4:**
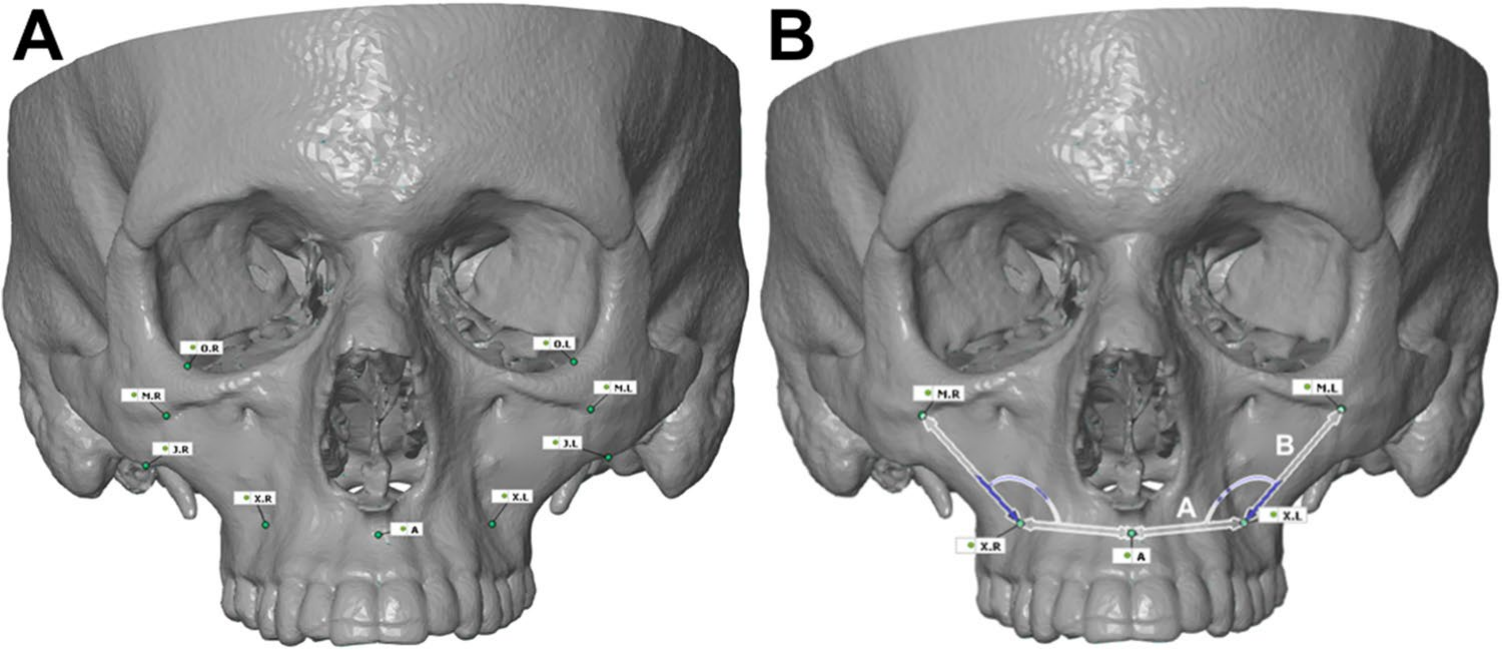
The relevant anatomical points of measurement for a standard hemimaxillectomy defect for a two-segmental reconstruction (image A). Values of interest are the length of the anterior segment (length **A**), the length of the posterior segment (length **B**) and the corresponding angle in between (∢AB) (image B)

The data generated was then used to address hemimaxillectomy defects for FFF reconstruction with two bony segments. To plan the segmental reconstruction, seven equally reproducible points had to be defined on both sides (Fig. 4). The first point was the A point (A) [19]. To construct the second and third points, the most caudal and lateral points of the zygomatic bone (J) had to be located, and the shortest connecting line to the orbital rim (O) had to be defined from this point. The exact center of these lines then determines the position of the points (M). For the next two points, the apex of the second maxillary premolar was marked and, if necessary, reconstructed by approximation (X). These points were then used to create a ‘2-point distance’. To do this, points M and X, and points X and A on both sides were connected. This created the defined distances A (AX) and B (MX). A ‘3-point angle’ could be constructed at the center point of these lines, describing the relationship between the legs of the angle and each other. All of these raw data was transferred for subsequent statistical analysis.

### Statistical analysis

For the statistical analysis, descriptive statistics were used to describe the data. An interferential analysis was then used to relate the data collected from the surface comparisons to patient age and sex. To avoid interrater bias, all 3D models and surface comparisons were created, analyzed and scored by one person. The software RStudio 2023.03.0 with R-4.2.1 was used for statistical analysis. Outliers were considered as 1.5* interquartile range (IQR) (normal outlier) and 3* IQR (extreme outlier), which is a liberal or conservative rule for labelling outliers [20]. The focus was on extreme outliers. On a descriptive level, the following distance measures were examined: Dmean, AVD, ID and IAD. Correlations are represented by the Pearson correlation coefficient (r). The Chi-square test was used to examine differences at the nominal level. Significant differences were examined using ANOVA (ANalysis Of VAriance) for more than two independent variables; otherwise, the Welch test was used for exactly two independent variables. Regardless of whether a normal distribution was present, both tests gave valid results [21–23]. Given that in most cases, more than one variable is present, multivariate normality would have to be tested, which poses statistical problems not solved yet [24]. Therefore, the presence of a normal distribution was not tested. At the inferential level, univariate k-means cluster analysis was used to identify the most consistent regions and interactions with the patient characteristics of sex and age. For cluster analysis this study relies on K-means clustering algorithm. Future studies may investigate the performance of varying algorithms such as DBSCAN. The parameters used for the cluster analysis were Dmean, ID and IAD. Regions were analyzed separately for these parameters. The Elbow method was used to determine the optimal number of clusters [25, 26]. The distances AX (A) and MX (B) and the angles between them were analyzed descriptively.

## Results

A total of 34 (68%) male and 16 (32%) female patients aged between 20 and 89 years were included in the study. The mean age of the patients was 55.68 years (Table 1). All patients had a normal maxillary region, and all CT scans were obtained during routine clinical diagnostics.

**Table 1:** Patient characteristics

		Overall (*n* = 50)
Age [years]	mean (median)	55.68 (58.7)
	range (min–max)	69 (20–89)
	SD	19.51
Sex [*n* (%)]	male	34 (68%)
	female	16 (32%)

Graphical representation of all 50 surface comparisons between the reference and patient skulls revealed some striking results. Region 2 represented the most homogeneous surface. Region 2 was mostly coded in green, as the deviation from the reference was a maximum of 1 mm in the dorsal or ventral direction. A grey area indicates that the deviation from the reference skull was greater than 5 mm; therefore, no comparisons could be made in these invalid areas. Most invalid areas were found in region 4, which included the left and right alveolar ridges. This area appeared to be the most heterogeneously colored region in comparison. Most of the red areas were located there, showing a deviation of 4–5 mm. It was also noticeable that region 1 had the most blue areas and was therefore, on average, located in front of the reference skull.

This study focused on extreme outliers (3*IQR), most of the extreme outliers occurred in region 4, and the fewest occurred in region 1. Regarding the demographic characteristics of gender and age, stratified by region, a significant effect was found for age in relation to region 4 using the Welch test (*t*(30.411) = 2.88, *p* < 0.01). Subjects with an extreme outlier value (*M* = 45.41, *SD* = 19.64) were younger than those without (*M* = 56.13, *SD* = 19.41). The effect was also significant but less pronounced for region 3, where the outlier group (*M* = 46.29, *SD* = 18.82) was younger than the sample average (*M* = 56.76, *SD* = 19.53). Females had more outliers than males for region 3 (*X*^2^ (1, *N* = 50) = 14.05, *p* < 0.001) and region 4 (*X*^2^ (1, *N* = 50) = 6.40, *p* < 0.01). No other differences were found for outliers. When examining the outliers, the extreme outliers for the AVD were strongly correlated with the regions that were judged to be incomplete (*r* = 0.85, *p* < 0.001). After removing the extreme outliers in the AVD, there was a significant difference (*p* < 0.001) between the side affiliation only for this parameter; for all other parameters and regions, no significant or relevant observations could be made. Based on this observation, the side affiliation (i.e., left or right side) of each region was not considered in further analyses (Table 2).

**Table 2:** Mean (M) and standard deviation (SD) for all regions and parameters of the surface comparisons of the patient skulls to the reference skull

Distance (M ± SD)	Region			
	1	2	3	4
Dmax	2.04 (1.42)	4.67 (0.39)	3.8 (1.79)	4.04 (1.14)
Dmin	‒4.35 (1.27)	‒4.88 (0.19)	‒4.15 (1.27)	‒4.59 (0.96)
Dmean	‒1.76 (0.88)	‒0.29 (0.61)	‒0.43 (1.7)	0.14 (1.09)
DSD	1.29 (0.4)	1.68 (0.37)	1.42 (0.46)	1.93 (0.56)
AVD	341.46 (16.98)	641.63 (54.69)	517.26 (61.21)	525.76 (12.77)
ID	‒612.84 (283.58)	‒149.09 (395.16)	‒271.73 (876.63)	55.74 (614.23)
IAD	703.8 (230.48)	913.82 (271.69)	939.51(420.2)	1006.36 (326.22)

*Dmax* Max Distance, *Dmin* Min Distance, *Dmean* Mean distance, *DSD* distance std deviation, *AVD* Area of valid distance, *ID* Integrated distance, *IAD* Integrated absolute distance

**Fig. 5:**
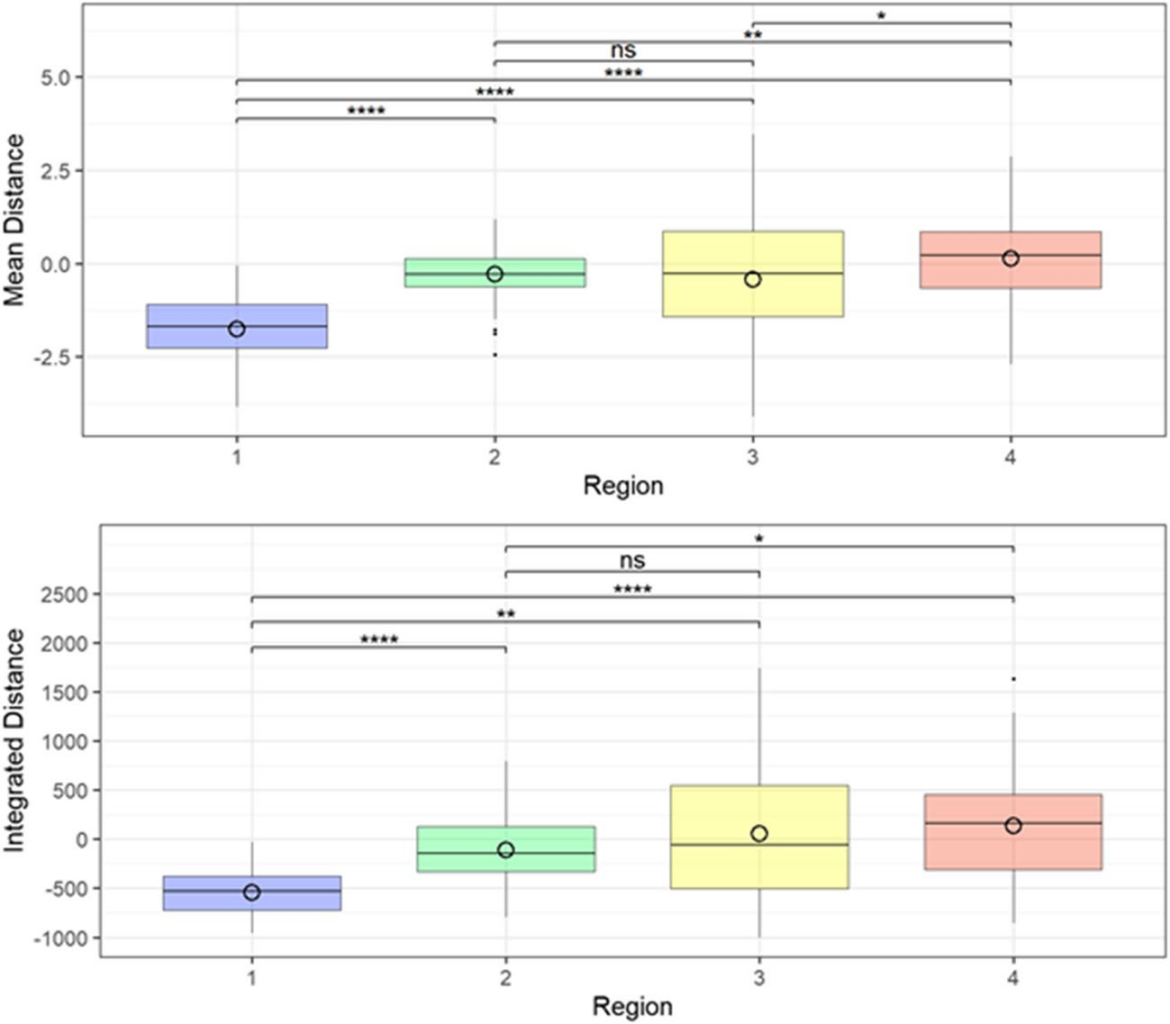
Results of the Mean distance (Dmean) [upper boxplot, y-axis in mm] and of the Integrated distance (ID) [lower boxplot, y-axis in mm^3^] per region

The following analysis of the differences between the regions focused on the parameters Dmean, AVD, ID and IAD. These data were analyzed using ANOVA tests and then are presented as box plots. Both significant (*) and non-significant (ns) differences were found between the regions. In region 2, both the dispersion around the median, the so-called interquartile range (IQR), and the difference between the minimum and maximum Dmean were the smallest, whereas in region 3, both were the largest. There was a significant difference between all regions, except between region 2 and region 3. The dispersion increased from regio 1 to region 2. Next larges IQR was found for region 4, with region 3 having the largest IQR aund subsequently the largest range between the maximum and minimum. As with the Dmean, the median was negative in regions 1, 2 and 3 and positive only in region 4. Most outliers were found in region 2 (Fig. 5).

Univariate K-means clustering was performed separately for the Dmean, ID and IAD parameters. In most cases, the Elbow method suggested the formation of two or three clusters. Interdisciplinary examination favoured the two-cluster solution. The analysis was designed to determine whether the patient characteristics of sex and age interacted with the groupings suggested by cluster analysis. However, few anomalies were found in this respect. A significance test was not performed to avoid HARKing ("hypothesizing after the results are known"); instead, the results are reported descriptively [27].

In summary, region 1 produced mostly negative values for both Dmean and ID. From these values it can be concluded that region 1 is on average located in front of the reference skull. It is noticeable that most male patients are in the negative value clusters, i.e. they tend to be larger than the reference skull, suggesting a gender-specific characterization of region 1. Region 2 is quite constant throughout the analyzed results, it has the smallest dispersion (IQR) for Dmean and it is the most homogeneous region in the graphical analysis.

Region 3 showed the greatest variation in Dmean, ID and IAD. In the graphical analysis there are more incomplete area comparisons, so the average completeness is lower than in the other regions. Overall, cluster analysis revealed a major influence of gender for region 3, with the majority of male skulls being in the negative value clusters for Dmean and ID, meaning that the patient skull is in front of the reference skull. This finding suggests a larger zygomatic anatomy.

Region 4, with the alveolar process, is strongly influenced by the patient’s dental status. Due to individual anatomy, region 4 was significantly different between the left and right sides. The medians for Dmean and ID were positive only in region 4, indicating that the region was posterior the reference skull. This finding suggests that the analyzed cohort had less volume on average compared to the fully edentulous reference skull due to tooth loss or bony atrophy. Region 4 is therefore the most difficult to assess and also showed the most extreme outliers (n = 15) for AVD, which had to be excluded from further analysis.

These results allowed further distance and angle analyses to be carried out. Following the area comparisons, the lengths of distances A (AX) and B (MX) and the '3-point angle' between them, were measured. For the right side, the mean angle was 132.06° (SD: ± 6.04°), while for the left side, it was 130.42° (SD: ± 4.95°). The sample size could be doubled if the difference between the sides was ignored. The combined mean angle was 131.24° (SD: ± 1.29°), which was positioned between the mean values of the individual right and left angles. For the results, the mean total distance B (MX) was 28.15 ± 0.86 mm, which is smaller than the mean total distance A (AX) of 30.71 ± 0.57 mm. The analysis of gender characteristics showed that the angle was larger on average in women than in men, with a larger angle indicating a more obtuse measurement. Conversely, both distance B (MX) and distance A (AX) were greater in the male group than in the female group. The difference in size between males and females is more pronounced for distance B (MX) at 5.48 mm than for distance A (AX) at 2.24 mm. The mean values of the male skulls (28.86 mm for distance B and 30.80 mm for distance A) are closer to the total mean values (28.15 mm for distance B and 30.71 mm for distance A) than those of the females. The total angle, at 131.24° ± 1.29°, is between the mean values of the male and female angles (Table 3).

**Table 3:** Measurement results for the reconstructive segments of a standard hemimaxillectomy defect

	left (*n* = 50)	right (*n* = 50)	overall (*n* = 100)
AB angle [° ± SD]	130.42 ± 4.95	132.06 ± 6.04	131.24 ± 1.29
A segment [mm ± SD]	28.07 ± 3.03	28.24 ± 3.07	28.15 ± 0.86
B segment [mm ± SD]	30.65 ± 2.06	30.78 ± 2.37	30.71 ± 0.57

A segment: A-point (deepest concavity of the alveolar process of the maxilla in the median plane) to apical first molar, B segment: apical first molar to midpoint zygomatic bone, AB angle: angle between segments A and B

## Discussion

The more complex spatial anatomy of the maxilla, compared to the mandible, necessitates more demanding reconstructive planning for upper jaw reconstructions. This complexity is a primary reason why VSP is often favored for maxillary reconstruction. This contrasts with the mandible, where there are more reconstructive alternatives between VSP and conventional surgery. The aim of this study was to conduct basic research to establish an intermediate reconstructive method for maxillary reconstruction between the conventional technique and VSP. Analogous to the established reconstructive options for the mandible [11], the aim of this study was to investigate whether a mean value-based, partially adjustable cutting-guide system is possible for the maxilla. To this end, it was necessary to anatomically analyze the maxilla with regard to segmental bone reconstruction and to assess its clinical feasibility. Due to the greater anatomical complexity of the maxilla in terms of existing angles and surfaces, it cannot be analyzed using the same methodology as that used for the mandible in the preliminary work [13]. In contrast to the maxilla, the most clinically relevant anatomy of the mandible is mainly seen in a two-dimensional plane. In this simplified view, the mandibular body has a parabolic shape, and it was therefore possible to divide the mandibular body into two-dimensional reconstructive segments according to the principle of segmental resection and reconstruction [3]. The lengths of the individual segments and the various angles between them were then analyzed, ultimately enabling the development of a partially adjustable cutting-guide system.

However, the maxilla does not have such clearly defined two-dimensional segments, but rather consists of curved surfaces that affect several planes. Therefore, it was not possible to perform an analogue measurement, but a three-dimensional surface analysis of the maxilla was required. Such an analysis, with a focus on bony reconstruction, has not been described in the literature and therefore had to be created. The necessary anatomical data were obtained from high-resolution CT scans, and the complex spatial structures of the maxilla were digitally analyzed using appropriate software tools. This method of measurement has been reported in the literature to be useful in the equally complex anatomical analysis of cleft lip and palate patients. In these cases, 5 mm surface comparisons were made to a constant reference in order to analyze the different anatomies of cleft lip and palate patients [28]. *Seidel *et al*.* also used a similar method to analyze soft palate tissue using surface comparisons [29]. In this study, the linear distance and volume between the gingival surface and the palatal bone were measured for each patient after cone beam computed tomography and intraoral scans. Another application of this method has been described for 3D analysis of the condylar position after different reconstructive approaches for mandibular reconstructions with FFF [30]. The deviation and rotation of the condylar heads before and after surgery were assessed in 3D by performing a surface comparison between the pre- and post-operative data sets.

The present analysis revealed gender and anatomical differences. Region 1 showed negative Dmean and ID values, indicating that it is generally anterior to the reference skull, with males predominantly in this category. Region 2 was the most consistent, with minimal variation and uniformity in Dmean. Region 3 had the greatest variation and least completeness, with males predominantly having negative values, indicating a larger zygomatic bone. Region 4 was influenced by dental status, with significant left–right differences and generally positive Dmean and ID values, suggesting less volume due to tooth loss or atrophy. It also had the most outliers. Our complex analysis of the maxillary anatomy suggested that the anatomical variance of the regions studies was suitable for semi-standardized segmental resection of the maxilla. Therefore, a further analysis of reconstructively relevant distances and angles was performed to prove the possibility of such a semi-standardized reconstruction.

Analogous to the digital analysis of the mandibular anatomy, distances and angles were measured for each patient and then analyzed together. The removal of the side affiliation is based on the assumption that a potential resection and reconstruction template system should be equally applicable to both sides. For this purpose, relevant points were defined on each patient’s skull, and sections A (AX) and B (MX) were constructed. The selection and anatomical localization of the defined distances A and B are adapted for possible two-segment reconstruction of maxillary defects. The corresponding distance and angle analysis showed that the angle on the right sides was 132.06°, the mean angle on the left side was 130.42°, and the combined angle was 131.24°. The distance B (MX) was smaller than that of A (AX), with larger values in males. The angles of the female were larger, while the distances of the males were closer to the overall mean.

There is limited published data on the measurement of distances and angles specifically related to FFF reconstruction of the maxilla. The literature focuses primarily on vertical and perpendicular measurements of the midface and maxilla [31, 32]. However, these measurements do not directly correlate with maxillary reconstruction using FFF. Therefore, our study represents the first attempt to collect data on surface comparisons of maxillary anatomy relevant to surgical reconstruction.

A limitation of the study is its uniqueness and therefore its lack of comparability. One influencing factor is the anatomical variability in the morphology of the patient cohort included. However, a heterogeneous database is also essential in order to be able to treat a large number of different patients with similarly diverse anatomy with potential reconstruction systems. There are certainly some other limitations due to the manual determination of anatomically surgically relevant surfaces. These were defined by the working group according to the Brown/Shaw classification of maxillary reconstructive units [16]. The clinically acceptable tolerance of 5 mm was also determined by the working group on the basis of surgical-reconstructive expertise. No comparative data for these two parameters can be found in the literature.

## Conclusion

Maxillary reconstructing is challenging due to its complex anatomy. The aim of this study was to provide anatomical data for the development of a partially adjustable cutting guide system for maxillary reconstruction, bridging the gap between conventional techniques and virtual surgical planning (VSP). High-resolution CT scans and digital tools revealed sex-specific and anatomical differences in four reconstructive regions of interest, although these differences did not have a clinically relevant impact. The anatomical findings of the study support a semi-standardized segmental resection approach. Relevant distance and angle analyses for two-segment FFF reconstructions of hemimaxillary defects showed that the combined mean angle between segments was 131.24°. The posterior segment distance B (MX) was smaller than that of A (AX), with both being greater in males. Despite its limitations, this landmark anatomical study is critical to the development of additional means-based semi-standardized surgical reconstruction systems that enable simple and cost-effective reconstructions. This study is intended to provide the basis for the development of a partially adjustable cutting guide system for maxillary FFF reconstruction.

## Data Availability

Most of the data generated or analyzed are included in the article. The remaining datasets used and/or analysed during the current study are available from the corresponding author on reasonable request.
